# The Nourishing Part of Meat

**Published:** 1898-07

**Authors:** 


### THE NOURISHING PART OF MEAT.

The only nutrient portion of meat is the solid part. Hence beef tea, although stimulating, has no food value. The only portion of the flesh of an animal which is possessed of real nutritive value is that part which has been alive and active before death. These living structures are not soluble; if they were, an animal which happened to fall into the water would dissolve like a lump of sugar. During life there is a small portion of nutritive material in solution in circulation in the body. After death, this small amount of soluble food material is rapidly converted into excrementitious matter; and as the skin, kidneys, and lungs cease their action, these poisonous substances rapidly accumulate within the body, the molecular or cell life of the body continuing some hours after death.

It thus appears that beef tea, as a French physician recently remarked, is a veritable solution of poisons. The only portion of the flesh which has any nutritive value is that which is thrown away in making the beef tea or extract. The popular faith in beef tea as a concentrated nourishment has, however, become so thoroughly fixed and rooted that some time will be required to rid the world of this erroneous idea; but it is highly important that information upon the subject should be disseminated as rapidly and as widely as possible, for there is no doubt that many lives are aunually sacrificed by faith in the superior nutriment value of meat juices.—*Public Health Journal.*

